# Supplementary figures and images for: Prognostic significance of C-reactive protein (CRP) and albumin-based biomarker in patients with breast cancer receiving chemotherapy
Source: PeerJ. 2025 May 21;13:e19319. doi: 10.7717/peerj.19319 (PMC12103165; doi:10.7717/peerj.19319)

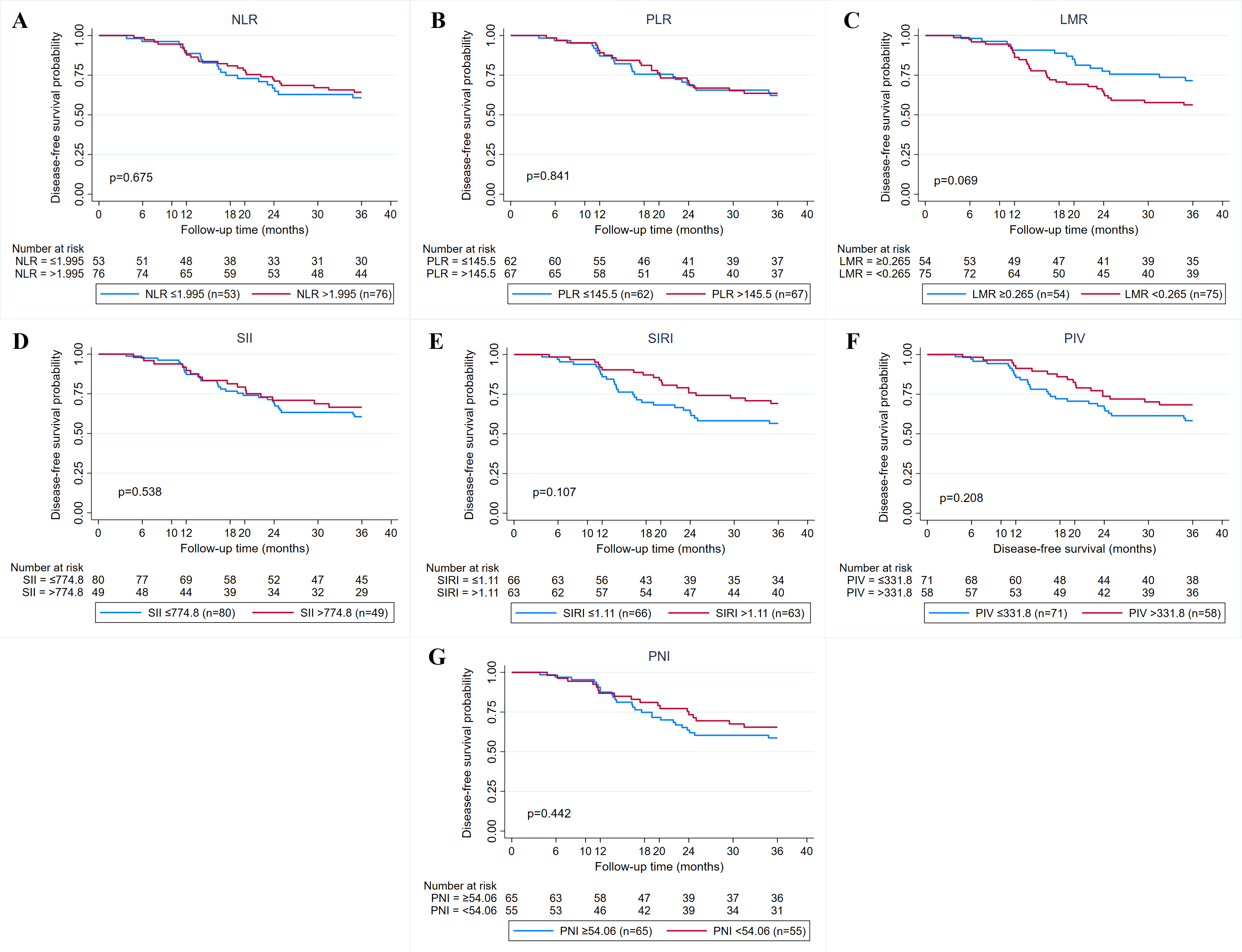

Supplement: Supplemental Information 2 — (A) Neutrophil-to-lymphocyte ratio (NLR). (B) Platelet-to-lymphocyte ratio (PLR). (C) Lymphocyte-to-monocyte ratio (LMR). (D) Systemic immune-inflammation index (SII). (E) Systemic inflammation response index (SIRI). (F) Pan-immune inflammation value (PIV). (G) Prognostic nutritional index (PNI). [file peerj-13-19319-s002.png]

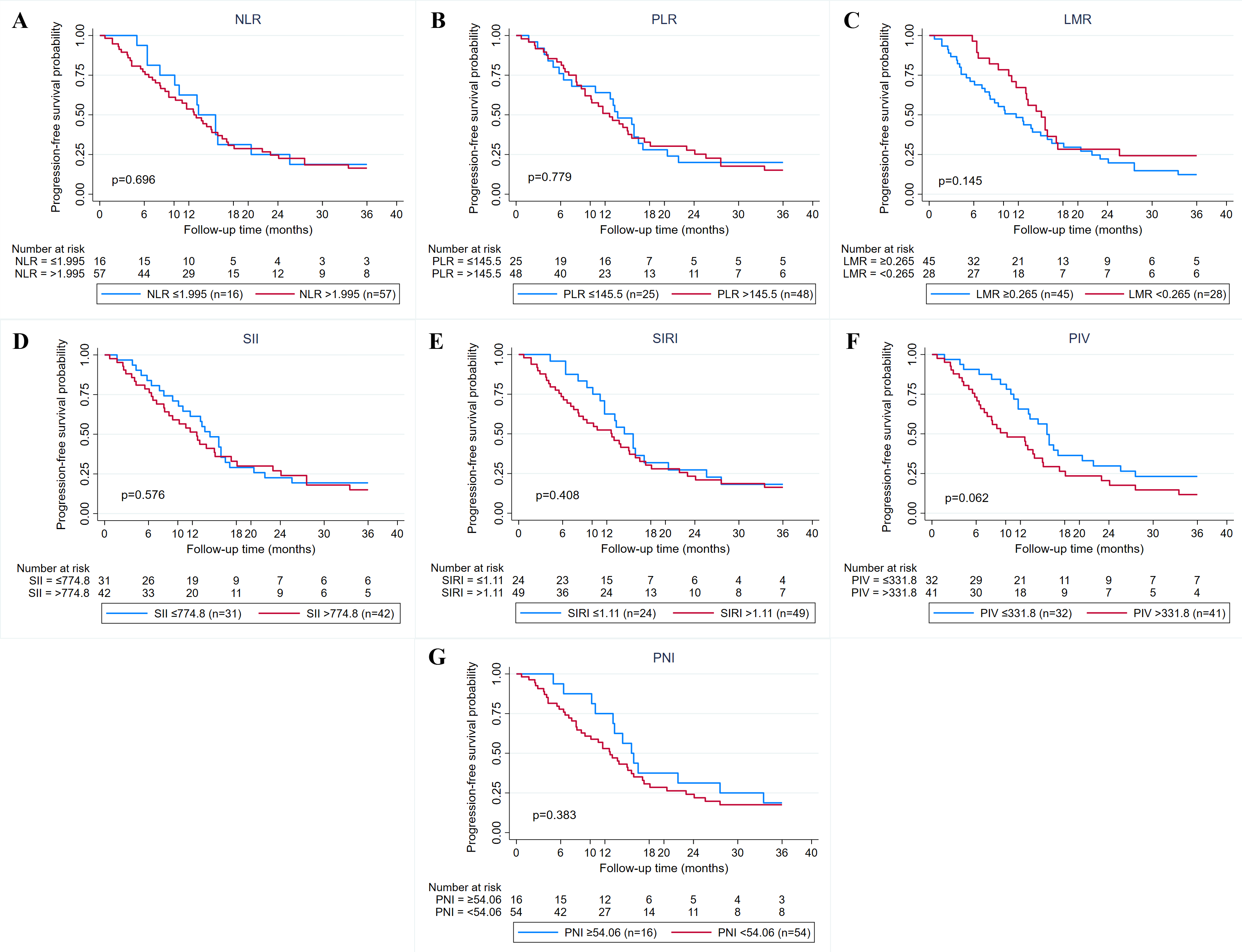

Supplement: Supplemental Information 3 — (A) Neutrophil-to-lymphocyte ratio (NLR). (B) Platelet-to-lymphocyte ratio (PLR). (C) Lymphocyte-to-monocyte ratio (LMR). (D) Systemic immune-inflammation index (SII). (E) Systemic inflammation response index (SIRI). (F) Pan-immune inflammation value (PIV). (G) Prognostic nutritional index (PNI). [file peerj-13-19319-s003.png]

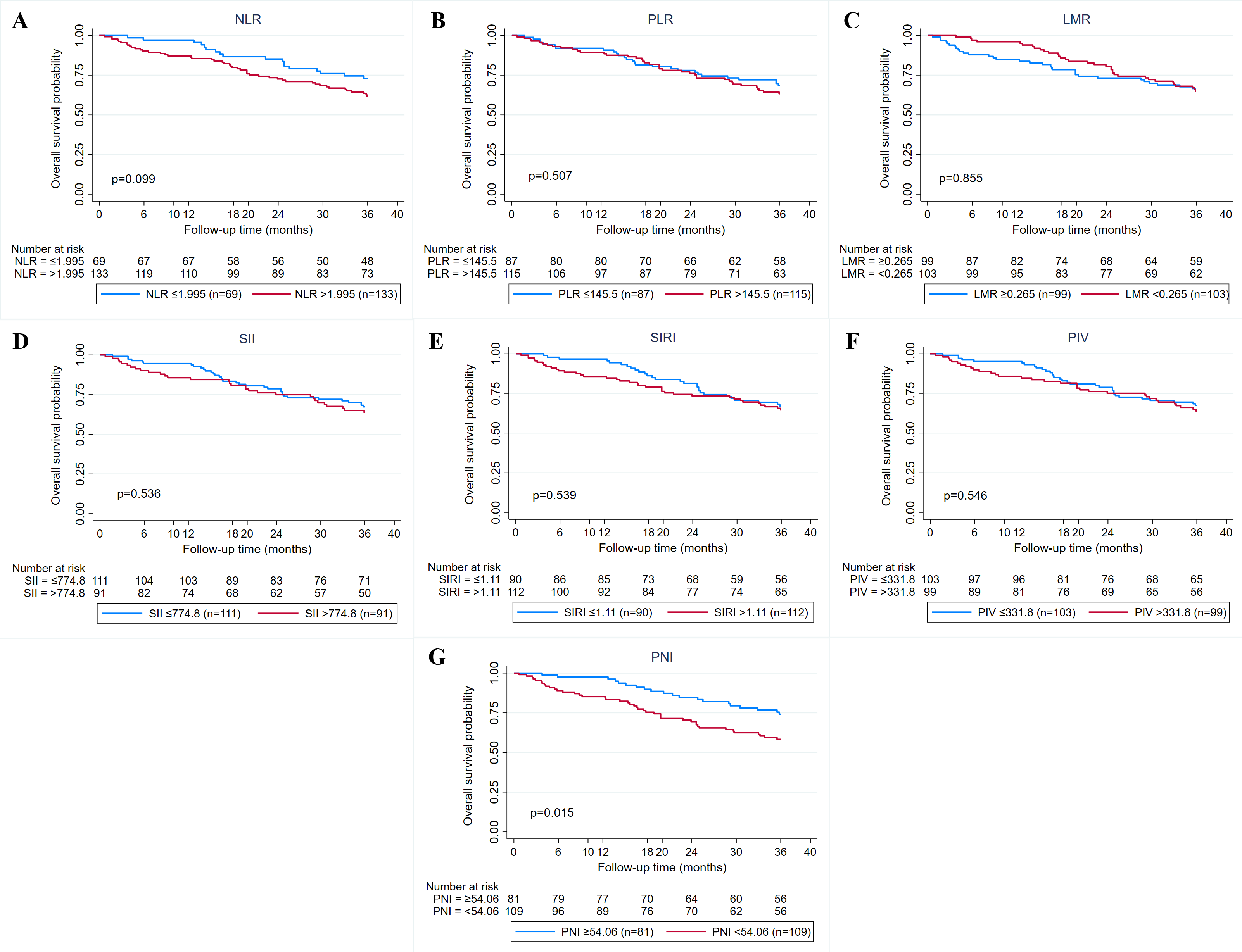

Supplement: Supplemental Information 4 — (A) Neutrophil-to-lymphocyte ratio (NLR). (B) Platelet-to-lymphocyte ratio (PLR). (C) Lymphocyte-to-monocyte ratio (LMR). (D) Systemic immune-inflammation index (SII). (E) Systemic inflammation response index (SIRI). (F) Pan-immune inflammation value (PIV). (G) Prognostic nutritional index (PNI). [file peerj-13-19319-s004.png]
